# Therapeutic relationships and the problem of containment: Experiences of patients at a psychiatric training hospital

**DOI:** 10.4102/sajpsychiatry.v25i0.1246

**Published:** 2019-10-24

**Authors:** Manfred W. Böhmer, Christa Krüger

**Affiliations:** 1 Department of Psychiatry, University of Pretoria, Pretoria, South Africa

**Keywords:** therapeutic relationships, containment, psychiatric inpatients, psychiatric hospital, psychiatry registrar training

## Abstract

**Background:**

The biopsychosocial model emphasises the role of human relationships in psychiatric care. Therapeutic relationships that improve treatment outcome and provide containment are desperately needed by patients in distress. Despite the importance of human relationships, they are neglected in an era dominated by biological psychiatry.

**Aim:**

This qualitative research project explores the experiences, perceptions and subsequent needs of patients. The role of therapeutic relationships, and the factors that patients felt influenced their relationship with their therapists, were examined.

**Setting:**

A psychiatric training hospital in South Africa.

**Method:**

Thirty in-depth semi-structured interviews were conducted with 15 inpatients. A qualitative, explorative-descriptive, collective case study design was used. Purposive sampling ensured maximum variation and richness of information. Grounded theory methods were used to analyse transcribed recordings.

**Results:**

Patients valued therapeutic relationships that provide containment and potentially obviate the need for ‘measures of control’. A model of containment was developed to demonstrate the various factors that interact in the attempt to provide containment to patients in a psychiatric training hospital system.

**Conclusion:**

Training hospitals should emphasise the role of therapeutic relationships in achieving containment and positive treatment outcomes. In developing countries, severe shortcomings in mental healthcare resources hinder the building of personal therapeutic relationships.

## Introduction

In his 1986 lecture to the American Psychiatric Association (APA), Reiser lamented registrars’ lack of curiosity about the patient as a person. He stated that most of the registrars would learn more about the stranger next to them on a short airplane trip than in formal psychiatric interviews.^1^ In 2001, Gabbard, another winner of the Educator Award of the APA, warned that reducing the provision of psychotherapy could reshape psychiatry in a biologically reductionistic direction.^2^ In addition, in 2012, Shapiro wrote that registrars have learnt to manage their patients instead of listening to them, and that the focus of modern psychiatry is not on relationships but on making a diagnosis from the Diagnostic and Statistical Manual of Mental Disorders (DSM) and prescribing medication.^3^

Engel introduced the biopsychosocial model four decades ago to overcome a narrow biomedical approach and to put the focus on the patient as a person.^4^ He emphasised that the work of the physician is done within an ongoing human relationship. More recently, the concept of person-centred psychiatry emerged, aiming to put the person and not the symptoms or illness at the centre of healthcare.^5^ A person-centred approach and the building of therapeutic relationships are important for various reasons. Therapeutic relationships not only improve engagement and satisfaction with healthcare services, but also improve adherence to medication and treatment outcomes.^6,7,8,9,10^ A biopsychosocial approach, combining medication with psychotherapy, leads to better results in a variety of conditions.^2^

Patients with mental illness need more than a narrow biomedical approach as they are often in distress and suffer from intense feelings, for example, despair,^11^ loneliness,^11^ or a fear of disintegrating.^12^ They need a personal relationship with someone whom they can trust and who is able to provide containment through listening, understanding and empathy, ‘a capacity to share in the experiences of others’ (p. 34),^13^ and is able to bear intense, distressing feelings.^14^

This study was conducted at Weskoppies Psychiatric Hospital, the main psychiatric training hospital of the University of Pretoria, Pretoria, South Africa. The main rationale for this study was the growing focus in psychiatry on a symptom-oriented and not person-oriented approach,^2,3,15^ and thus the concern about the experiences of patients about care and treatment provided and the implications for the training of registrars.

The South African Society of Psychiatrists (SASOP) reported in a recent media statement that the problem of care, treatment and training is worsened and complicated by the ‘appalling state of mental health services’ in South Africa.^16^ Mental health services are underfunded, facilities are in a dire state and a severe shortage of psychiatrists exists.^17,18^ For example, in the period up to 2010, KwaZulu-Natal province had only 0.38 psychiatrists per 100 000 population in the public sector.^17^

We explored different aspects of experiences and needs of patients and the types of interaction between registrars and inpatients in an open and broad way. The main outcomes of this research were the development of a model of personal interaction and containment in a psychiatric training hospital system.

## Method

A qualitative, explorative-descriptive, collective, instrumental case study was conducted. The study received ethical clearance from the Ethics Committee of the Faculty of Health Sciences, University of Pretoria. A participant information leaflet and informed consent form were developed and the participants were informed about the objectives of the study. It was explicitly stated that non-participation would not affect their treatment in any way and that they could opt out from the study at any time.

### Setting and sampling

The study population comprised patients admitted to Weskoppies Hospital for general psychiatric services.

We sampled purposively and for maximum variation to ensure that all population groups were represented.^19,20^ Inclusion criteria for the study were as follows: the ability to speak English or Afrikaans to exclude the need for an interpreter; participants who had expressed specific positive or negative experiences regarding treatment to their treating team; patients who were admitted for an extended period of treatment, or with whom their team identified specific problems relating to relationships; and male and female patients of different race, backgrounds and diagnostic groups. Patients were only included if their consultant psychiatrists were of the opinion that the patients were stable enough to participate and they could express themselves coherently in an interview. Exclusion criteria included an inability to sustain a conversation because of severe mental illness.

Saturation was reached after 30 in-depth semi-structured interviews were held with 15 patients.^20,21,22^

Eight female and seven male patients, aged 24–62 years, were interviewed. Their level of education varied from Grade 2 to university degrees. Patients were, on average, admitted for 12 weeks. Five patients suffered from bipolar I disorder, two from bipolar II disorder, two from major depressive disorder, one from schizophrenia, one from schizoaffective disorder, one from adjustment disorder and three from substance-induced disorders.

The group of participants altogether had been seen by 12 different registrars during their present hospitalisation, and some had seen other registrars during previous admissions. Weskoppies Hospital had 18 registrars at the time of the study (2014-2015). Registrars rotate through the various training stations on a 4-monthly basis; these include general adult psychiatry, forensic psychiatry, child- and adolescent psychiatry, community clinics, one peripheral hospital and neurology.

There are five multidisciplinary teams (MDTs) for adult services in Weskoppies Hospital, each comprising a consultant psychiatrist, a consultant psychologist, two or three registrars, two intern psychologists, two social workers and an occupational therapist.

### Collection and management of data

The first author, who was not involved in the treatment of the participants, conducted all the in-depth interviews. A questionnaire guided the interviews. The interview guide covered the following areas: experiences of patients in the hospital, in different types of wards, with the registrars, type and quality of relationships that developed and with whom, how these relationships affected the outcome of treatment, the care received, participants’ opinion about the training of the staff member and with whom they would like to follow up and why. The questionnaire was developed in a pilot study and further expanded in the first few interviews. The interviews lasted up to 70 min and were conducted in a private room in patients’ wards. The interviews were conducted in English or Afrikaans to obviate the need for an interpreter and resultant loss of confidentiality. Of the 30 interviews, 25 were audiotaped and 22 were fully transcribed for analysis. Non-transcribed interviews were conducted to clarify statements received in initial interviews. Field notes were made of all interviews, hospital files were studied and observations about participants were noted.

### Analysis

The first author managed and analysed the qualitative data using ATLAS.ti software. Data were analysed using grounded theory methods.^22,23,24^ Open, axial and selective coding were used to group the data into categories and core themes, which were refined through the process of constant comparison.^22,23,24^

### Establishing trustworthiness

Measures used to establish trustworthiness included reflexivity (the process by which the researcher reflects on the data collection and interpretation process), audit trail (careful documentation of all aspects of the research, including field notes, audio recordings, transcriptions, etc.), triangulation (testing validity through the convergence of information from different sources) and crystallisation (temporarily suspending the process of immersion in the data to reflect on the analysis experience), thick descriptions (paying attention to contextual detail in observing and interpreting social meaning) and member validation (sharing parts of the findings with research participants), according to recognised principles of qualitative research.^22,23^ As an additional example of reflexivity, the first and second authors held regular weekly discussions lasting for about 1 h to reflect on the research process. As an example of triangulation, the second author coded four interviews separately to help reflect on the way the coding was done.

### Ethical consideration

Ethical clearance was obtained from the Research Ethics Committee of the Faculty of Health Sciences, University of Pretoria (reference number 354/2013). All study participants provided written informed consent prior to their participation in the study.

## Findings

From the patients’ perceptions and experiences, the main theme that emerged was how important the doctor-patient interaction and therapeutic relationship are in the treatment planning and in helping patients cope with their distress and anguish. Such a relationship helped patients feel safe and contained. Important in developing this relationship was the interaction with individual members of the MDT and also between the individual members of the MDT and the MDT as such. The following are some selected quotations to illustrate these themes.

### Feeling distressed and in need of more personal contact and interaction

A 30-year-old female patient, studying for a master’s degree in the arts and recovering from a manic episode, described feeling helpless about the decision-making process regarding her treatment:

‘Helpless, ja. Helpless, because the doctors are the ones who decide. … there is nothing you can do.’ (Int. 18)

A 32-year-old single male patient, with highest qualification Grade 12, was still in a locked ward but had recovered from a psychotic episode. He felt uncertain about his treatment plan and questioned whether he could trust his registrar to keep her promises:

I ‘Doctor X is in the ward now, has she seen you today?’

P ‘No, not yet.’

I ‘When will she see you again?’

P ‘I don’t know, because she said she was going to see me again next week. But I don’t know whether today she is going to see me or not. Because I’m really not sure of that.’ (Int. 6)

Most of the participants had been admitted to locked wards for varying periods. Admissions to locked wards were described as very difficult, especially if patients were put into seclusion. A 46-year-old single woman, with a bachelor’s degree in arts, who was secluded during a manic episode, described it as follows:

‘It is hell. Life can be hell. Life can be hell. At times you feel: God are you still there, are you still there …’ (Int. 19, [translated from Afrikaans])

An elderly lady, in her second marriage for 29 years and a Grade 10 qualification, was immediately admitted to a locked ward following an outburst of anger:

I ‘What had happened when you broke the window of the psychologist?’

P ‘He carried on about my son’s death [*due to suicide*] and the more I said, I do not know, the more he said, but I should know. When he repeated that again, I became furious; I initially wanted to hit him, but I then broke the window. Then they locked me up.’ (Int. 9, [translated from Afrikaans])

A 44-year-old man, who had dropped out of university, had resigned from his job and had a history of several admissions, echoed the need for more personal contact:

‘New doctors only ask how you are, do you sleep well, are you hearing voices – that type of stuff … They should familiarize themselves with the history of the patient … If they don’t, you already know that this is not really going to help.’ (Int. 2, [translated from Afrikaans])

Of the 15 participants, only a small minority of three described good to very good relationships with their registrars. The above quoted 32-year-old single man, who had been hospitalised for 5 weeks, drew attention to this problem. He only found meaningful and helpful interactions with fellow patients:

I ‘But would you say that there was a specific person, one person, with whom you developed a meaningful interaction?’

P ‘No, I wouldn’t say that.’

I ‘But you told me about those patients, the fellow patients …’

P ‘Yes, to me it was the most meaningful interaction … because … you’re able to talk to a person who’d really understand your situation and would give you enough chance to explain yourself and that is more meaningful I think …’

I ‘How do you think that affected you, that you did not have such a therapeutic relationship [*with a team member*]?’

P ‘I think it has blocked me from growing. It has not allowed me to grow or to even venture enough to other possibilities that are, like, could be underlying in my condition that I could sort out and be able to deal with, because there’s a lot that is not being said, that I haven’t spoken about.’ (Int. 7)

### The importance of relating and connecting

Having recovered from a psychotic episode, the 32-year-old single man already quoted above, talked about the importance of being treated as a fellow human being:

I ‘I found it interesting that you said these trainee nurses that came in treated you as equals, as not someone who is sick, but just as a fellow human being.’

P ‘Yes … as trainees they were more like people who were not exposed so much to this kind of environment. … [*they were*] able to blend with us and not look at us as patients, but look at us as fellow human beings, because they were able to learn from us as well and we were able to learn from them. And they were able to take the knowledge that they got from us and utilise it to better their careers, I think.’

I ‘It sounds as if they approached you in a very open way, not with preconceived or preformed ideas.’

P ‘Exactly.’

I ‘And that was very meaningful and helpful.’

P ‘In a big way … in a big way. It really meant a lot to me because they were not judging us, yeah … they were there asking us questions like normal people, not like people who are mentally ill or something like that, but normal people. … They respected us.’ (Int. 5)

The same patient valued being able to teach a staff member something; it valued him as a human being:

‘I even taught one male nurse basketball. There was a basketball here, so I’m a very big fan of basketball and I play basketball a lot. So, I even taught that one guy how to score and he managed to be able to learn from me and he said to me, he’s going to try and practice as he goes home; whenever he gets a ball he’s going to keep on practicing. So, for me it was an eye opener that somebody can take something like that from me, it was good to … it was more like a highlight as well.’ (Int. 5)

Building a personal, therapeutic relationship will take time. The same patient is again quoted, describing the lack of therapeutic relationships with registrars – if someone really wants to know a patient, he or she must be a psychologist according to the patient:

P ‘They [*the registrars*] lack certain things …’

I ‘Like what?’

P ‘… With the doctors, they don’t have enough time … some patients they have a lot of stories to tell, or they need to be asked certain questions … the time that is given probably is small … and that would be not enough for some people. I guess that I think, that is lacking to the doctors. I think … whatever the doctors are trained for, they’re quick in doing things, like as I’m talking to you right now, I’m open, I’m speaking to you, and to me you sound like a psychologist, somebody who wants to know me, like, more.’ (Int. 5)

When asked how long it takes to build a therapeutic relationship, the 46-year-old patient, who had a degree in arts and lived with her mother and who had seen numerous registrars during her admissions for a bipolar disorder, had this to say:

P ‘It [*the therapeutic relationship*] can be established in the wink of an eye.’

I ‘In the wink of an eye?’

P ‘In the wink of an eye.’ (Int. 20, [translated from Afrikaans])

The patients who had good therapeutic relationships with their registrars, appreciated their registrars’ interest in them and willingness to listen. A 43-year-old married flight attendant, who had two sons, had this to say about the positive, non-verbal messages from her young, female registrar:

‘I didn’t want to speak about things that maybe bothered me. But the manner she approached the situation, made you want to speak about something …’ (Int. 22)

In the midst of loneliness or hopelessness a therapeutic relationship is of great importance:

I ‘What made her [*the registrar*] important?’

P ‘… I think it’s because she’s been with me on this road ever since last year … she knows my history… every single time I get to see her I see a bit of light.’ (Int. 22)

In the following quote, an elderly well-qualified man, who was divorced, had lost his job and home and was suffering from a major depressive disorder, expressed an intense need to keep up the connection with his registrar:

‘I would desperately like to follow up with Doctor M, I don’t want to meet the new doctor right now. I suspect it will be Doctor Y, but I would dearly love to be able to … discuss things with him [*with Doctor M*].’ (Int. 11, [translated from Afrikaans])

The importance of connections of having someone to talk to was highlighted by the fact that fellow patients became important to 10 of the 15 participants:

‘Yes, fellow patients, they have been most supportive and they’ve been amazing to me.’ (Int. 5)

It was surprising that for the 30-year-old female patient studying for a master’s degree in arts, a cleaner became one of the two most important people during her admission. There was a connection and a deeper sense of understanding – one had lost a husband, the other a son:

I ‘The cleaners? Do you speak more to the cleaners than to the sisters?’

P ‘Ja … one specific cleaner … she speaks the same language as me and she also lost her husband at the same month that I lost my son. So, we feel connected.’ (Int. 18)

A 32-year-old woman, in the middle of a divorce and studying for a degree in law, emphasised the interaction with two nursing sisters, linked to their ability to listen:

I ‘Do these two sisters have special qualities that made it easier for you?’

P ‘I think they show you respect and they know you are going through a difficult time … they take your feelings into consideration … there is immediately a connection … because you know they will listen to you …’ (Int. 13, [translated from Afrikaans])

Medical students also became important to the above well-qualified elderly man:

‘… and then, strangely enough, there were also medical students. The one brought two magazines for me yesterday. She is in her sixth year and a real gem.’ (Int. 11, [translated from Afrikaans])

### The interaction with the multidisciplinary team

Patients made many positive comments about being treated by an MDT. A few examples are:

The above 44-year-old man, who had dropped out of university, had resigned from his job and had a history of several admissions, said the following:

I ‘What stood out in your treatment? What was really meaningful?’

P ‘The fact that you are being treated by a professional team. It is not only your psychiatrist [*registrar*]; it is your social worker, your occupational therapist, your psychologist, and every one of them had a positive contribution to make.’

(Int. 1, [translated from Afrikaans])

The above 30-year-old female patient, studying for a master’s degree in arts, had the following to say:

I ‘Is there something positive about the team approach?’

P ‘Ja, there are positives. I think whatever decision this consultation, it’s different consultative, it simply means it’s not individualistic, whoever makes decisions has consulted with multiple consultants.’

I ‘And is there something positive in that?’

P ‘There should be, because I think the decision is more stronger than the individual.’ (Int. 18)

The above 32-year-old single male patient, with highest qualification Grade 12, said:

P ‘Good care is … you’ve mentioned the five people [*registrars, psychologists, occupational therapists, social workers and nursing staff*] that play a role in the lives of people that are patients here … they need to be known by the patients. The patient needs to know that I have five different elements that can be used in making my life better, you know, so then make use of them. If they don’t see a doctor, they wouldn’t mind that much, they would wish to see, probably, a psychologist, or the other ones that you have mentioned, a social worker if the person feels this problem’s more of work … I mean, home related you will see a social worker, yes.’

I ‘So good care would mean to involve all of those people.’

P ‘I think that would be good care, yes.’ (Int. 5)

## Discussion

Feelings of anxiety and uncertainty are common in patients admitted to psychiatric hospitals.^25,26^ In this study, patients often felt uncontained and suffered from intense distress. This was made worse by being admitted to locked wards and a lack of meaningful personal interactions with members of the MDT. For nearly all participants, relating and relationships were of great importance. Participants used emotionally laden and intense language to describe this. Patients emphasised having time, sharing, talking, listening, understanding and connecting. They felt more able to cope if they had this type of support. If registrars were not available, relationships were sought with others, for example, with fellow patients. Relationships were furthermore described as fostering growth and a sign of mental health.

Therapeutic relationships help provide the containment – a feeling of being held together and of being safe – needed by patients in distress or at risk. Containment can be attained through ‘measures of containment’ or through personal holding.^13,14,27^ ‘Measures of containment’, or rather measures of control, include, for example forced medication or admission to a locked ward and contrast with containment provided through a personal form of holding.^13,14,27^ The latter refers to a person being available to help with difficult feelings, a person having the emotional capacity to bear feelings too difficult for the patient to manage on his or her own. Medication can subdue such feelings, but what is really needed is a form of emotional holding such as a mother gives to her distressed child.^13^ Containment and holding, central elements of a therapeutic relationship, provide the secure base that makes treatment possible.^14^ Measures of containment or control should only be used when this fails.

If staff members are only experienced as being positive and supportive when a patient is healthy, this may indicate a lack of tolerance and containment. Tolerance and containment are especially needed when life is too much to bear.

To help create a safe space that provides containment and understanding of the patient, keeping to a frame linked to boundaries is essential. Psychiatrists working in a private practice have to provide this. The psychiatric registrar, average age 28–30 years, is less experienced and works in a system. This system comprises an MDT led by a consultant psychiatrist who fulfils, among others, a supervisory role. The registrar and the MDT constitute part of a bigger system, influenced by hospital management and by external factors such as the state of mental healthcare in a country. Patients emphasised the value they saw in being treated by an MDT.

**FIGURE 1:**
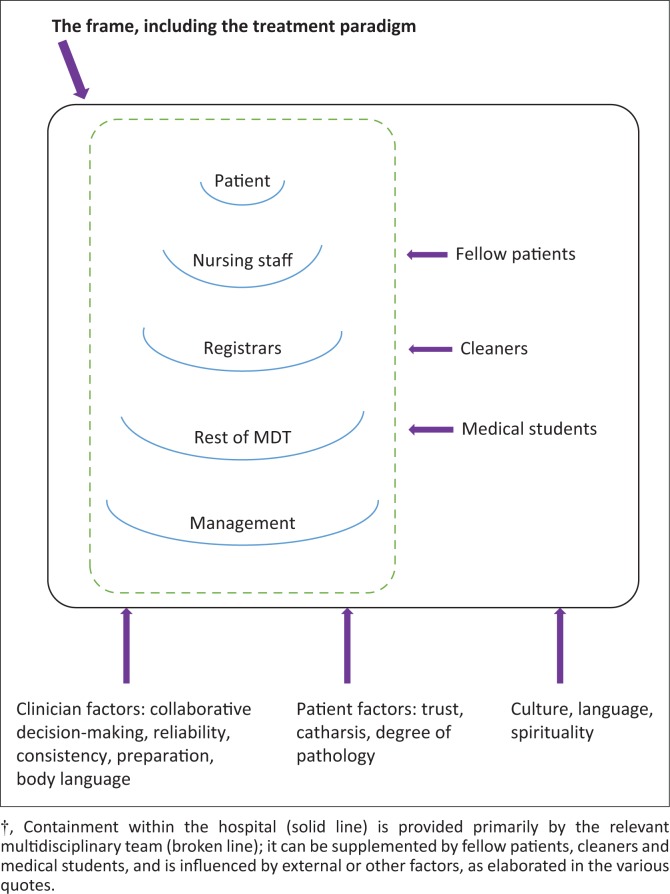
A model of containment in a psychiatric training hospital system.?

The factors influencing treatment and containment are illustrated in Figure 1, where the MDT and the rest of the hospital system are illustrated. We believe that an important starting point is whether the hospital advocates and follows a person-centred, biopsychosocial treatment paradigm as part of a treatment frame (solid line). Containment of the patient depends primarily on containment provided by the member of the MDT (broken inner line) primarily responsible for the patient who is able to build a therapeutic relationship with the patient. This member needs the support and cooperation of the rest of the MDT (represented by the various semi-circles). One interesting fact is that within the hospital environment, fellow patients, cleaners and medical students can be secondary sources of containment.

The MDT has become part of modern psychiatry. Having members of different professions available to patients will, however, not in and of itself necessarily improve care.^28^ It might, for example, happen, as in our experience, that certain members may feel intimidated by ‘experts’ in the team and may not voice their opinion. The team thus has to consider and protect the various working alliances between different team members and patients. In doing this, we believe that the MDT can help provide a containing space for therapeutic relationships.

**FIGURE 2:**
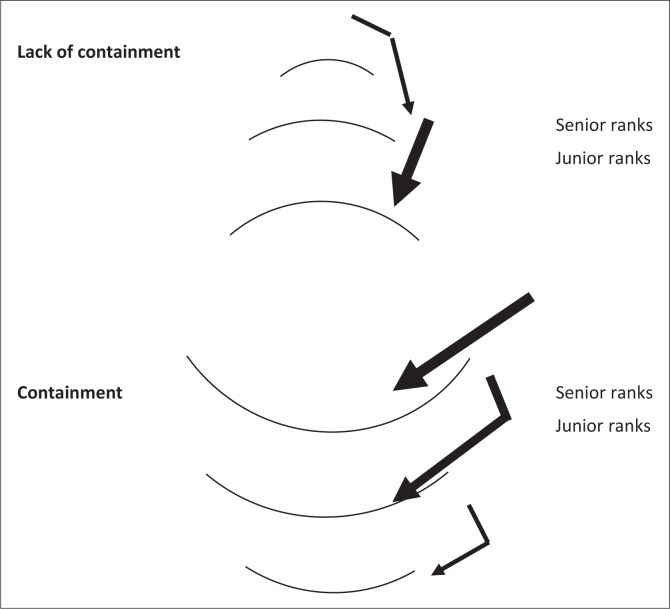
The interaction between senior and junior ranks in terms of providing containment: Absorbing stressors or deflecting stressors ‘downwards’.

Figure 2 depicts how we believe the interaction between senior ranks (government, management and consultants) and junior ranks (registrars and intern psychologists) might influence the ability to provide containment. Containment at senior levels should help and facilitate containment of patients by the junior ranks, while a lack of containment at senior levels can be expected to deflect stressors downwards and put junior ranks under pressure, resulting in a possible lack of containment to patients.

Are therapeutic relationships possible in situations of overwhelming need and time constraints? In this context, one participant answered that a therapeutic relationship could be established ‘in the wink of an eye’. The well-known psychoanalyst, McWilliams, writes that establishing a therapeutic relationship depends on several factors such as the clinician’s ability to have empathy and the patient’s degree of underlying psychopathology.^15^ Taking these variables into account, establishing a therapeutic alliance might be possible within minutes or could take years.^15^ Even in difficult situations, patients will however register the general attitude of the doctor and respond accordingly.^29^

*Alles wirkliche Leben ist Begegnung* [all real life is meeting, is personal engagement], writes Buber (p. 15).^30^ This study shows that patients need encounters or connections to others to feel supported and they will search out persons with whom this is possible. In developing countries, severe shortcomings in mental healthcare resources can hinder the building of personal therapeutic relationships. Notwithstanding such challenges, all psychiatric training hospitals should emphasise the role of therapeutic relationships in achieving containment and positive treatment outcomes.

### Limitations

This study was conducted in only one hospital and 15 participants reflected on the involvement of only 12 of the 18 registrars. It would be helpful to conduct similar studies in other training hospitals in South Africa and also other countries. A further limitation of the study was the fact that interviews were conducted only in English or Afrikaans, which meant that some of the patients could not participate in their mother tongue. This limitation had been weighed up against the loss of confidentiality that may come with the use of an interpreter and the potential loss of meaning and loss of therapeutic closeness when communication happens through a third person.

## Conclusion

Suffering from mental illness can be a frightening experience. Having a therapeutic relationship with an understanding healthcare provider, who is there to offer help and containment, can be life-saving. Containment provided through a personal relationship is, where possible, much more helpful than being subjected to ‘measures of control’, although such measures are at times unfortunately unavoidable. We developed a model of containment to demonstrate the various factors interacting in providing containment to patients in a psychiatric training hospital system. In addition to a call for government to attend to the needs of the mental health sector, the clinical implication is the need for a stronger focus on therapeutic relationships in the training of registrars.

## References

[1] Reiser MF Are psychiatric educators ‘losing the mind’? Am J Psychiatr. 1988;145(2):148–153. 10.1176/ajp.145.2.148 3341460

[2] Gabbard GO, Kay J The fate of integrated treatment: Whatever happened to the biopsychosocial psychiatrist? Am J Psychiatr. 2001;158(12):1956–1963. 10.1176/appi.ajp.158.12.1956 11729008

[3] Shapiro ER Management vs. interpretation. Teaching residents to listen. J Nerv Ment Dis. 2012;200(3):204–207. 10.1097/NMD.0b013e3182487a3e 22373756

[4] Engel GL The clinical application of the biopsychosocial model. Am J Psychiatr. 1980;137(5):535–544. 10.1176/ajp.137.5.535 7369396

[5] Mezzich JE, Salloum IM, Cloninger CR, et al Person-centred integrative diagnosis: Conceptual bases and structural model. Can J Psychiatr. 2010;55(11):701–708. 10.1177/070674371005501103 21070697

[6] Roche E, Madigan K, Lyne JP, et al The therapeutic relationship after psychiatric admission. J Nerv Ment Dis. 2014;202(3):186–192. 10.1097/NMD.0000000000000102 24566503

[7] Krupnick JL, Sotsky SM, Simmens S, et al The role of the therapeutic alliance in psychotherapy and pharmacotherapy outcome: Findings in the National Institute of Mental Health Treatment of Depression Collaborative Research Program. J Consult Clin Psychol. 1996;64(3):532–539. 10.1037/0022-006X.64.3.532 8698947

[8] Mintz DL, Flynn DF How (not what) to prescribe: Nonpharmacologic aspects of psychopharmacology. Psychiatr Clin N Am. 2012;35:143–163. 10.1016/j.psc.2011.11.009 22370496

[9] McKay KM, Imel ZE, Wampold BE Psychiatrist effects in the psycho-pharmacological treatment of depression. J Affect Disord. 2006;92:287–290. 10.1016/j.jad.2006.01.020 16503356

[10] Del Canale S, Louis DZ, Maio V, et al The relationship between physician empathy and disease complications: An empirical study of primary care physicians and their diabetic patients in Parma, Italy. Acad Med. 2012;87(9):1243–1249. 10.1097/ACM.0b013e3182628fbf 22836852

[11] Van den Berg JH A different existence. Pittsburgh: Duquesne University Press; 1972.

[12] Winnicott DW Fear of breakdown In: Winnicott C, Shepherd R, Davis M, editors Psychoanalytic explorations. London: Karnac Books, 1989; p. 87–95.

[13] Casement P On learning from the patient. London: Tavistock Publications; 1985.

[14] Holmes J Exploring in security. Towards an attachment-informed psychoanalytic psychotherapy. London: Routledge; 2010.

[15] McWilliams N Psychoanalytic diagnosis: Understanding personality structure in the clinical process. 2nd ed. New York: The Guilford Press; 2011.

[16] South African Society of Psychiatrists (SASOP) Media statement. S Afr Psychiatr. 2017;12:82–83.

[17] Burns JK Mental health services funding and development in KwaZulu-Natal: A tale of inequity and neglect. S Afr Med J. 2010;100(10):662–666. 10.7196/SAMJ.4100 21080996

[18] Gillis L The historical development of psychiatry in South Africa since 1652. S Afr J Psychiatr. 2012;18(3):78–82. 10.4102/sajpsychiatry.v18i3.355

[19] Nieuwenhuis J Qualitative research designs and data gathering techniques In: Maree K, editor First steps in research. Pretoria: Van Schaik, 2007; p. 69–97.

[20] Fossey E, Harvey C, McDermott F, et al Understanding and evaluating qualitative research. Aust N Z J Psychiatr. 2002;36:717–732. 10.1046/j.1440-1614.2002.01100.x 12406114

[21] Guest G, Bunce A, Johnson L How many interviews are enough? An experiment with data saturation and variability. Field Meth. 2006;18:59–82. 10.1177/1525822X05279903

[22] Cresswell JW Qualitative inquiry and research design: Choosing amongst five approaches. 3rd ed. Thousand Oaks, CA: Sage; 2013.

[23] Schurink W, Fouché CB, De Vos AS Qualitative data analysis and interpretation In: De Vos AS, Strydom H, Fouché CB, et al., editors Research at grass roots. 4th ed. Pretoria: Van Schaik, 2011; p. 397–424.

[24] Richardson L, St. Pierre EA Writing: A method of inquiry In: Denzin NK, Lincoln YS, editors The SAGE handbook of qualitative research. 3rd ed. Thousand Oaks, CA: Sage, 2005; p. 959–978.

[25] Bowers L, Haglund K, Muir-Cochrane E, et al Locked doors: A survey of patients, staff and visitors. J Psychiatr Mental Health Nurs. 2010;17:873–880. 10.1111/j.1365-2850.2010.01614.x 21078002

[26] Haglund K, Von Essen I Locked entrance doors at psychiatric wards – Advantages and disadvantages according to voluntary admitted patients. Nordic J Psychiatr. 2005;59:511–515. 10.1080/08039480500360781 16316906

[27] Bowers L, Alexander J, Ryan C, et al Cultures of psychiatry and the professional socialization process: The case of containment methods for disturbed patients. Nurse Educ Today. 2004;24(6):435–442. 10.1016/j.nedt.2004.04.008 15312952

[28] Swartz L Culture and mental health: A Southern African view. Cape Town: Oxford University Press; 1998.

[29] Böhmer MW Does psychiatry need religion and spirituality in its treatment approach? Narcissism as an example. S Afr J Psychiatr. 2016;22(1):a563 10.4102/sajpsychiatry.v22i1.563 PMC6137964 30263149

[30] Buber M Ich und Du In: Buber M, editor Das dialogische Prinzip. 5th ed. Heidelberg: Lambert Schneider; 1984.

